# Reversine enhances the conversion of dedifferentiated fat cells into mature cardiomyocytes

**DOI:** 10.12688/f1000research.122788.3

**Published:** 2024-06-11

**Authors:** Budi Baktijasa Dharmadjati, Djanggan Sargowo, Aulanni’am, Budi Susetyo Pikir, Yudi Her Oktaviono, Oryza Sativa, Kandita Arjani, Ricardo Adrian Nugraha

**Affiliations:** 1 Doctoral Programme in Biomedical Sciences, Faculty of Medicine, Brawijaya University, Malang, East Java, 65145, Indonesia; 2 Department of Cardiology and Vascular Medicine, Faculty of Medicine Universitas Airlangga, Dr. Soetomo Academic General Hospital, Surabaya, East Java, 60286, Indonesia; 3 Department of Cardiology and Vascular Medicine, Faculty of Medicine, Brawijaya University, Malang, East Java, 65145, Indonesia; 4 Biochemistry Laboratory, Department of Chemistry, Faculty of Sciences, Brawijaya University, Malang, East Java, 65145, Indonesia

**Keywords:** Cardiomyocyte, cTnT, DFAT cells, GATA4, Reversine

## Abstract

**Background:**

There is an essential need for cardiomyocyte regeneration among patients with heart failure. Transplantation of dedifferentiated fat (DFAT) cells may lead to an improvement of cardiomyocyte regeneration among heart failure patients. We believe that DFAT cells are promising candidate cell sources for cardiac regeneration. However, the pathway underlying how DFAT cells of the adipose lineage differentiate into mature cardiomyocytes isn’t fully understood.

**Methods:**

We conducted an experimental laboratory study on isolated DFAT cells from adipose tissue of healthy adults. Then, we treated cells with different concentrations of reversine (10, 20 and 40 nM), and performed RNA extraction and cDNA synthesis. Next, we used a ceiling culture method based on the buoyancy properties of mature lipid-filled adipocytes. Stemness expression (Octamer-binding transcription factor 4 [Oct4], brachyury, Fetal liver kinase 1 [Flk-1]) was quantified by reverse transcription-quantitative (RT-q)PCR, while cardiomyocyte expression (Transcription factor GATA-4 [GATA4] and cardiac troponin T [cTnT]) was quantified by immunocytochemistry.

**Results:**

ANOVA with Tukey’s post-hoc found that 10 nM reversine increased greater Flk-1 expression compared to the control group (MD: 5.037 + 0.998; *p* < 0.001), but there were no significant changes among Oct4 (MD: 0.013 + 1.244; *p* = 0.99) and brachyury expression (MD: 0.157 + 0.084; *p* = 0.252). Kruskal-Wallis revealed that the expression of GATA4 (1.65 [0.41-1.98] to 0.015 [0.007-0.034]; *p =*0.017) reduced significantly from day 7 until day 21 and cTnT (5.07 [6.62-8.91] to 8.22 [6.81-9.40]; *p*= 0 *.001)* increased significantly from day 7 until day 21.

**Conclusions:**

Reversine could increase the expression of Flk-1, but it was unable to stimulate the expression of Oct4 and brachyury related to cell stemness. An optimal concentration of 10 nM reversine may have the greatest effect on enhancing the differentiation of DFAT cells into mature cardiomyocytes, as indicated by higher cTnT expression between cells.

## Abbreviations

ACE: Angiotensin Converting Enzyme

ASCs: Adipose derived Stem Cells

BMSCs: Bone Marrow Mesenchymal Stem Cells

CMC: Cardiac Mesodermal Cell

CPC: Cardiac Progenitors Cell

cTnT: Cardiac troponin T

DFAT: Dedifferentiated Fat Cell

END-2: Endoderm-like cell line

ESCs: Embryonic Stem Cells

EPCs: Endothelial Progenitor Cells

FBS: Fetal Bovine Serum

Flk-1: Fetal Liver Kinase 1

HAT: Histone Acetylation Transferase

HeSCs: Human Embryonic Stem Cells

hMSC: human Mesenchymal Stem Cell

HSCs: Hematopoietic Stem Cells

HSC: Hepatic Stellate Cell

iPSC: Induced Pluripotent Stem Cells

MEK: Mitogen activated extra cellular signal regulated kinase

MPC: Mesodermal Progenitor Cell

MPS1: Mono-polar Spindle 1

MSC: Mesenchymal Stem Cell

μMM: Non-Muscle Myosin II

Oct4: Octamer-binding transcription factor 4

PBS: Phosphate Buffer Saline

PSC: Pluripotent Stem Cell

SVF: Stromal Vascular Fraction

TMSC: Tonsil Mediated Stem Cell

UVSCs: Umbilical Vein Stem Cells

## Introduction

Heart failure is a complex health problem due to its high morbidity and mortality, so a definite and efficient approach to heart failure is a long-term hope and goal in the development of heart disease therapy. The population of those with heart failure increases as the number of older individuals grows and life expectancy generally increases due to the advances in pharmacology, interventions, and cardiac surgery. Classic interventions focus on controlling factors that might worsen cardiac function. These interventions do not solve the real problem, because widespread and progressive cardiomyocyte dysfunction has still taken place. Thus, novel approaches that modify the remodelling process, focus on the regeneration of cardiomyocytes, and improve the performance of cardiomyocytes are required. ^1^

Regenerative medicine combines an interdisciplinary research and clinical application that aims to repair, replace, and/or regenerate cells, tissues and/or organs to restore disturbed function. ^1^^,^^2^ These approaches include, but are not limited, to the use of small and/or soluble molecule, gene therapy, stem cell-based therapy, organ tissue engineering, and reprogramming of cells. ^3^^,^^4^ As a precursor for stem cell harvesting in cell-based therapy, adipose tissue has unique advantages in terms of abundant availability, ease of isolation, high degree of homogeneity, and decent ability to differentiate into multipotent cells. ^5^^,^^6^ The identification of an ideal cell source for tissue regeneration remains a challenge in the stem cell field. ^7^ With the current development of isolation and culture techniques, ceiling culture and insert culture will produce adipose-derived stem cells (ASCs) with a higher quantity of dedifferentiated fat (DFAT) cells. ^8^^,^^9^

Similar to ASCs, DFAT cells have been proven to be able to differentiate into various cells in the mesenchymal lineage. ^10^^,^^11^ DFAT cells have plasticity to differentiate into several cell types derived from the three germ layers, including skeletal myoblasts, ^12^ vascular endothelial cells, ^13^ neurons, ^14^ and urethral smooth muscle cells. ^15^

DFAT cells are a population of cells which can be safely harvested from patients. Due to their low immunogenicity and reported benefits, they are already being recognized as approved therapeutic product. They were demonstrated to be safe but their effects were not always consistent, as preclinical studies suggested. ^7^ This may be due to poor survival in disease environments and/or their limited ability to differentiate into other cell types, which is important for the processes of tissue reconstruction and tissue engineering and has clinical, biochemical or molecular implications. ^7^

Reversine is a purine-derived small molecule that has been shown to be able to induce the dedifferentiation of unipotent myoblast cells to progenitor cells, which are multipotent. ^16^^,^^17^ Reversine as an inductor of cell dedifferentiation that has the ability to increase the potency of a cell, so that the cell can be directed to differentiate into another cell in the same lineage or even from a different lineage with exposure to the appropriate stimulus and medium. ^18^^,^^19^

To date there has been no research that proves the effect of reversine on the process of further dedifferentiation of adipocyte cells, which causes a reduction in the commitment lineage so that it can be relatively more easily directed towards target cells, so this study aims to analyse the effect of reversine exposure and differences in passages of DFAT cells related to their differentiation into cardiomyocytes, characterized by the expression of the marker Transcription factor GATA-4 (GATA4) and cardiac troponin T (cTnT). We performed a pilot study to understand the optimal concentration of reversine to maximise the potency of DFAT cell dedifferentiation into cardiac progenitor stem cells. We used different concentrations of reversine (10, 20 and 40 nM) in the DFAT cell cultures.

## Methods

### Ethical approval

Our study obtained a letter of approval from Health Research Ethics Council of Faculty of Medicine, Universitas Airlangga (reference number: 062/EC/KEPK/FKUA/2021) issued on March 22 ^nd^ 2021, under the name of Budi Baktijasa Dharmadjati as the principal investigator. All procedures were approved by the relevant ethics committees, and written informed consent was obtained from all study participants.

### Study design

The study design is an observational, analytical laboratory study using different concentrations of reversine in DFAT cell cultures from human adipose tissue from a 39 years-old woman. This type of analytical laboratory study is a post-test only controlled group design. The independent variables consist of various concentrations of reversine (10, 20, 40 nM). The dependent variables consist of the expression of cell stemness biomarkers (Octamer-binding transcription factor 4 [Oct4], brachyury, Fetal liver kinase 1 [Flk-1]), early cardiomyocytes (GATA4) and mature cardiomyocytes (cTnT).

### Study setting

Our research was conducted at Institute of Stem Cell Laboratory Airlangga University, Suraaya, East Java and Dr. Soetomo General Academic Hospital, Surabaya, East Java. The duration between study enrolment until data completion lasted for 12 months.

### Number of replications

The number of replicates that will produce this interval half-length according to Berthouex and Brown (2002) ^20^ is:

n=Zα2σE2

This formula assumes random sampling. It also assumes that n is large enough that the normal distribution can be used to define the confidence interval. Then, the number of replications for each group is four, so the total sample needed is 16 replications.

### Materials

1. DFAT cells from adipose tissue were collected from the lower abdominal area of patients during laparoscopic surgery with small incisions (3-5 cm) under local anaesthesia by a digestive surgeon. Cells were collected from adult patients who were in a stable condition and were not taking anti-platelets or anti-coagulants. Patient was prepared for clinical application of stem cell therapy at the Network Bank Dr. Soetomo General Hospital, Surabaya. DFAT cells were multiplied *in vitro* at the 3 ^rd^ and 6 ^th^ passage.
2. Reversine (2-(4-morpholinoanilino)-6-cyclohexylaminopurine) is an Aurora kinase inhibitor that is able to dedifferentiate DFAT cells into mesodermal stem cells. The control group (P0) had four culture cells and were not treated with reversine. The treatment groups (REV10, REV20, REV40) included 12 culture cells treated with different concentrations of reversine (10, 20 and 40 nM).
3. Washing buffer containing phosphate-buffered saline (Sigma-Aldrich, Milan, Italy), 0.1% sodium azide (Sigma-Aldrich, Milan, Italy), and 0.5% bovine serum albumin (BSA) Sigma-Aldrich, Milan, Italy) was used for all washing steps (3 ml washing buffer and centrifugation, 400 × g for 8 minutes at 4°C). Briefly, 5×10 ^5^ cells/sample were incubated with 100 ml of 20 mM ethylene-diaminetetraacetic acid (EDTA, Sigma-Aldrich) at 37°C for 10 minutes and washed.
4. Proliferation media with α-Minimum Essential Medium (MEM) (Sigma-Aldrich, Milan, Italy), which contains non-essential amino acids, sodium pyruvate, lipoic acid, vitamin B12, biotin, and ascorbic acid.
5. Differentiation media with CDM3-C medium (with CHIR99021) (Sigma-Aldrich, Milan, Italy).
6. Transport media with 3% gentamicin (Thermo Fisher Scientific, Inc., Waltham, MA, USA)
7. Type 1 Collagenase (Worthington Biochemical Corporation, Lakewood, NJ, USA)
8. Triple express, trypan blue and PSC Cardiomyocyte Differentiation Kit (Gibco, Thermo Fisher Scientific, Inc., Waltham, MA, USA)
9. Mouse IgG Monoclonal Antibody against Cardiac troponin T Recombinant (1:300 dilution; LSBio (LifeSpan) Cat# LS-C89784-200, RRID:AB_1936961) and F(ab')2-Goat anti-Rabbit IgG (H+L) Cross-Adsorbed Secondary Antibody, Alexa Fluor™ Plus 488 (Thermo Fisher Scientific Cat# A48282, RRID:AB_2896345)
10. Anti-Rabbit GATA4 Polyclonal Antibody (1:100 dilution; Thermo Fisher Scientific, Inc., Waltham, MA, USA: Catalog # BS-1778R) and F(ab')2-Goat anti-Rabbit IgG (H+L) Cross-Adsorbed Secondary Antibody, Alexa Fluor™ Plus 488 (Thermo Fisher Scientific Cat# A48282, RRID:AB_2896345)
11. 2 mL aspiration pipettes individually wrapped (Greiner, cat. no. 710183)
12. 5, 10, 25 mL pipettes sterile, individually wrapped (Corning Falcon, cat. nos. 357543, 357551, 357535)
13. 250-, 500-, and 1,000-mL PES 0.2 μm filters (Thermo Scientific Nalgene, cat. no. 568-0020, 569-0020, 567-0020)
14. 100 μm cell strainer (Corning Falcon, cat. no. 352360)
15. 0.6 mL sterile microtubes (E&K Scientific, cat. no. 280060-S)
16. 2 mL sterile microtubes (E&K Scientific, cat. no. 280200-S)
17. 100 mL glass beaker (Fisher Scientific)

### Experiments

*Collection and isolation of DFAT cells from adipose tissue*

Adipose tissue obtained from loose subcutaneous tissue through abdominoplasty procedure with a size of ~3x3 cm with an estimated weight of 10 g and stored in a 50 mL conical tube. Adipose tissue was minced into small pieces and dissociated with 0.1% (w/v) collagenase, then sent to the laboratory using a container containing an ice pack at a temperature of 4°C without the addition of other cryopreservation agents. When the tissue was received in the laboratory, the adipocyte tissue was put into a 50 mL plastic tube to be washed with D-PBS (-) twice at room temperature. The tissue was then placed into a 10 cm glass petri dish, then chopped until smooth.

Insert culture strategy was performed to collect mature lipid-filled adipocytes based on the property of buoyancy. Approximately 1 g adipose tissue was chopped and mixed into 0.1% collagenase solution (type I collagenase) at 37°C for 1 hour with stirring/shaking slowly. After centrifugation at 135 × g for 3 minutes, the supernatant layer containing DFAT cells floated on the culture medium or plastic tube to allow other non-adipocytes to separate and sink to the bottom and can be discarded after centrifugation, then filtered using a nylon filter (core size 100 m). Adipose was then washed repeatedly (3 times) in MEM supplemented with 20% foetal bovine serum (FBS) before further use. A total of 30 µL was then transferred to 6-well plates with 70 µm filters and incubated for 5 days in MEM. DFAT cells from adipocytes will sink through the filter and stick to the bottom of the dish. The filter and residual adipocytes were discarded after day 5.

Cells were treated with different concentrations of reversine (10, 20 and 40 nM). Reversine (StemCell Technologies) at a dose of 10, 20 and 40 nM was administered to DFAT cell sub-groups passage 3 and 6 on the first day of DFAT cells entering the passage. Then, DFAT cells were incubated for 15 minutes at room temperature.

*RNA extraction and cDNA synthesis*

For extraction of RNA, single cell pellets from each well in the first group were transferred to a conical tube for further centrifugation at 13,000 rpm for 3 minutes (Wisespin CF10, WISD). The pellets formed were separated and 200 μL liquid was left in the tube. Pellets were rinsed with PBS, then 560 µL prepared Buffer AVL containing carrier RNA (QiAmp ^®^ Viral RNA Kit) were added into a 1.5 mL microcentrifuge tube. Vortexing (Wisemix VM-10, WISD) was performed for 15 seconds followed by spin down (Vision VS-100 BN) before incubation at room temperature for 15 minutes. After the incubation process was completed, 600 μL of 96% ethanol was added to the sample tube. Vortexing was again performed for 15 seconds followed by spin down. The solution in the tube was then transferred to a spin column and centrifuged at 8,000 rpm for 1 minute. After the supernatant layer was removed, the contents of the tube were transferred to a 2 mL collection tube. Wash buffer AW1 was added to the collection tube before centrifugation at 8,000 rpm for 1 minute. After rinsing with wash buffer 2, the supernatant layer was removed and centrifuged at 12,000 rpm for 1 minute in order to dry the filter, with a target concentration in the filter of 1-2 ng/μL. Then the solution was transferred to a 1.5 mL tube and 60 μL AVE buffer was added. After incubation at room temperature for 3 minutes, centrifugation was carried out at 8,000 rpm for 1 minute. The solution was then stored in a collecting tube in a cooler at -20°C.

For cDNA synthesis, the RNA samples and reagents were thawed at room temperature and the PCR tubes were prepared and labelled. Vortexing was carried out to ensure that the solution mixture was homogeneous, and a spin down process was carried out to ensure that all liquids remained at the bottom. A total of 2 µL reagent was added to the PCR tube, then 3 µL RNA sample was added to the same PCR tube. After vortexing and spin down, the next tube was inserted into the PCR machine at 70°C for 5 minutes. cDNAs were generated from 1 mg total RNA and amplified using the ReverTra Ace qPCR-RT Kit (Toyobo, Osaka, Japan) according to the manufacturer’s instructions.

*Reverse transcription-quantitative (RT-q)PCR*

Quantification of cell stemness expression (Oct4, brachyury, Flk-1) was performed *via* RT-qPCR. Primer sequences of Oct4, brachyury and Flk-1 can be seen in Table 1. Primer reagent (0.5 μL) with nuclease-free water (1.5 μL) were vortexed and span down at 70°C for 5 minutes. Another reagent consisted of 4 μL Go Script buffer, 4 μL MgCl _2_, 1 μL PCR Nucleotides, 1 μL Reverse transcriptase, 0.5 μL RNA sin and 4.5 μL nuclease-free water. A concentration of 7.5 pmoles/μL primer reagent was resuspended with the primer pairs in 50 μL autoclaved deionized water and 0.1X TE buffer (1 mM Tris HCl, pH 8.0 at 25°C; 0.1 mM EDTA, pH 8.0 at 25°C). After the PCR reaction process was completed, the cDNA samples were stored in a refrigerator at -20°C. Expression was quantified using the 2 ^-ΔΔCt^ method and the fold-difference of expression levels of genes were calculated and compared in cycle threshold (Ct) values. ^21^ The melting curve was generated immediately after amplification by holding the reaction mixture at 95°C for 60 seconds, and then lowering the temperature to 45°C at a transition rate of 0.1°C/second and maintained for 120 seconds. Then, the samples were heated slowly at a transition rate of 0.05 to 80°C with continuous collection of fluorescence at 640 nm.

**Table 1.** Sequences of primer pairs for reverse transcription-quantitative PCR.

Primers	Primer sequences	Size (bp)
Oct4	F 5’-CCTGAAGCAGAAGAGGATCACC-3’ R 5’-AAAGCGGCAGATGGTCGTTTGG -3’	144
Brachyury	F 5’-CCTCGAATCCACATAGTGAGAG-3’ R 5’-AAGAGCTGTGATCTCCTCGT-3’	117
Flk-1	F 5’-ATGCACGGCATCTGGGAATC-3’ R 5’-GTCACTGTCCTGCAAGTTGCTGTC-3’	573

F, forward; R, reverse; Oct4, Octamer-binding transcription factor 4; Flk-1, Fetal liver kinase 1.

*Immunocytochemistry*

Quantification of cardiomyocyte expression (GATA4 and cTnT) was performed by immunocytochemistry. Assessment of the expression of the differentiation marker of GATA and cTnT in DFAT culture at this stage using the FITC-labelled immunocytochemical method, was carried out on days 7, 14 and 21 after the cells were exposed to differentiation medium. DFAT cells were incubated with a target unmasking fluid (Accurate Chemical & Scientific Corp., Westbury, NY, USA) for 15 minutes using a microwave oven for antigen retrieval.

The slides were reconstituted in PBS, pH 7.4 and blocked with 10% normal goat serum (v/v). For double staining, the slides were incubated with Anti-Rabbit GATA4 Polyclonal Antibody (1:100 dilution; Thermo Fisher Scientific, Inc., Waltham, MA, USA: Catalog # BS-1778R) and Mouse IgG Monoclonal Antibody against Cardiac troponin T Recombinant (1:300 dilution; LSBio (LifeSpan) Cat# LS-C89784-200, RRID:AB_1936961) at 4°C overnight and then washed three times with PBS. Some of the sections were incubated with 1% BSA as negative controls. Next, the sections were incubated with F(ab')2-Goat anti-Rabbit IgG (H+L) Cross-Adsorbed Secondary Antibody, Alexa Fluor™ Plus 488 (Thermo Fisher Scientific Cat# A48282, RRID:AB_2896345) respectively, for 1 h at 25°C (room temperature).

This was followed by incubation with DAPI (Vector Laboratories). The incubated monolayer cells were fixed with 4% formalin buffer for 15 minutes, then the object glass was washed with PBS and dried. Followed by blocking with 10% PBS for 15 minutes. Immunocytochemistry in the DFAT preparation was intended to observe the expression of cTnT where the cells were then incubated with fluorophore-labelled secondary antibody with F(ab')2-Goat anti-Rabbit IgG (H+L) Cross-Adsorbed Secondary Antibody, Alexa Fluor™ Plus 488 (Thermo Fisher Scientific Cat# A48282, RRID:AB_2896345). The staining process was carried out in a dark room because it was sensitive to light, then incubated at 37°C for 1 hour and then observed using an excitation filter of 450–560 nm under a fluorescence microscope (Nikon) at a magnification of 200×.

### Data analysis

We analysed the association between various concentrations of reversine (10, 20 and 40 nM) with the expression of cell stemness biomarkers (Oct4, brachyury, Flk-1 gene expression), and early cardiomyocyte (GATA4) and mature cardiomyocyte (cTnT) markers from the 6 ^th^ cell passage. Firstly, we did a normality test using Shapiro-Wilk test. For normally distributed data, we performed a comparison test using Analysis of Variance (ANOVA) with Tukey’s post-hoc analysis. For data that was not normally distributed, we performed Kruskal Wallis followed by a Mann-Whitney U test. Path analysis was carried out using multiple linear regression to determine the pathway mechanism of the influence of the concentration of reversine on the dedifferentiation of DFAT cells and differentiation into mature cardiomyocytes. Statistical tests were performed using IBM SPSS Statistics (RRID:SCR_016479) version 25.0 software.

## Results

### Characterization of DFAT cells as mesenchymal stem cells

**Figure 1.**
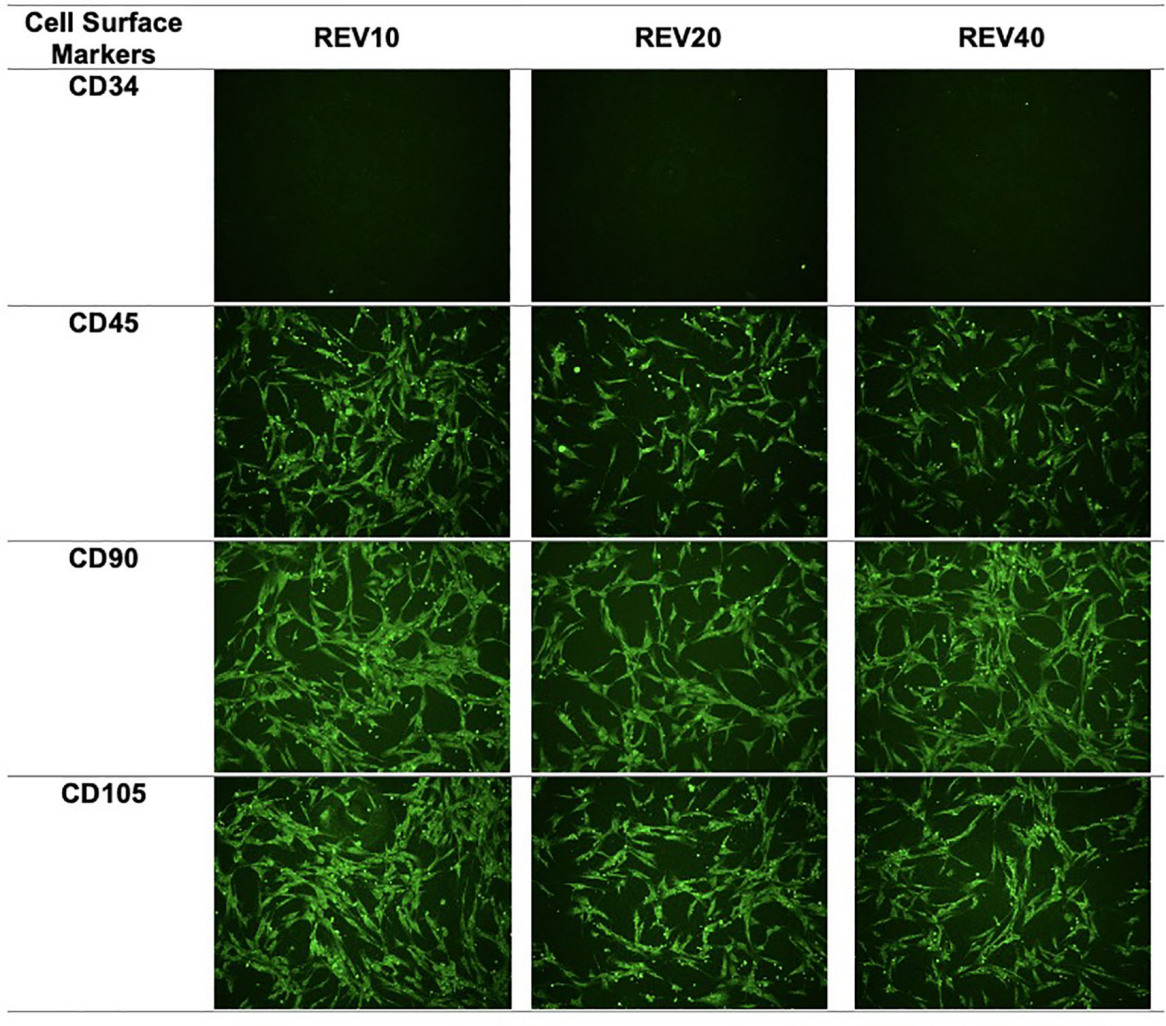
Immunocytochemistry images of mesenchymal stem cell surface markers. REV, reversine.

The surface markers used in the DFAT cell characterization process were CD90 and CD105, which are specific markers for mesenchymal stem cells, and CD45 and CD34, which are specific markers for hematopoietic cells. Examination by immunocytochemistry showed that the DFAT cell population showed positive expression (>95% luminescence of cells per field of view) of CD105 and CD90 and negative expression (<2% luminescence per field of view) of CD45 and CD34 ( Figure 1). ^22^^–^^24^

### Analysis of RT-qPCR for gene expression of DFAT (Oct4, brachyury, and Flk-1)

DFAT cells were analysed using the RT-qPCR method on days 3, 5, and 7. The mean data from the experiment from the 6 ^th^ cell passage is in Table 2. As seen in Table 2, the REV10, REV20, and REV40 groups had a lower mean value of Oct4 gene expression compared with group K. Similarly, for the mean expression of brachyury gene expression, all reversine groups had lower values than the control group. The opposite occurred for Flk-1 gene expression, where reversine groups always have higher mean value than control group, with REV10 and REV40 had the highest value of Flk-1 gene expression. Analysis using the ANOVA test showed that there were significant differences in the mean values of gene expression (Oct4, brachyury, and Flk-1) at each reversine dose (REV10, REV20, and REV40) as can be seen in Table 2.

**Table 2.** Mean value and ANOVA for Oct4, Brachyury, and Flk-1 gene expression based on the concentration of reversine.

	Group	Mean	SD	Mean Square	Sig. (ANOVA)
Oct4	Control	1.00	0.00	1.839	<0.001
	REV10	0.98	0.39		
	REV20	0.62	0.45		
	REV40	0.17	0.94		
Brachyury	Control	1.00	0.00	1.878	<0.001
	REV10	0.84	0.18		
	REV20	0.37	0.35		
	REV40	0.15	0.96		
Flk-1	Control	1.00	0.00	99.574	<0.001
	REV10	1.03	0.89		
	REV20	1.01	0.00		
	REV40	1.03	0.26		

Oct4, Octamer-binding transcription factor 4; Flk-1, Fetal liver kinase 1; REV, reversine.

**Figure 2.**
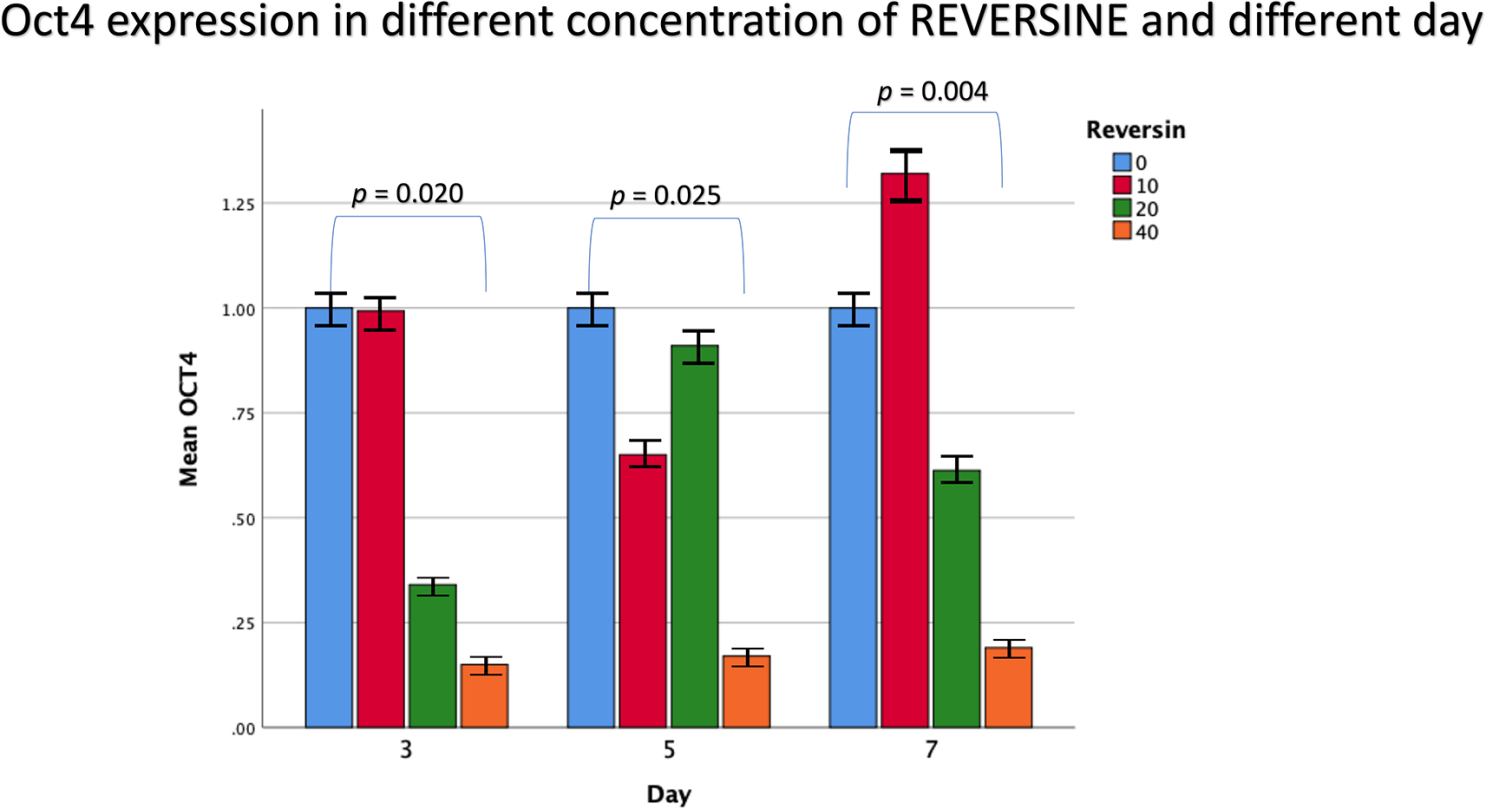
Graph showing that Oct4 gene expression is highest in the control group, and decreased in the REV10, REV20 and REV40 groups, except on day 7. Oct4, Octamer-binding transcription factor 4; REV, reversine.

The mean value for Oct4 gene expression from the 6 ^th^ cell passage showed a decreasing trend with the addition of reversine. There was a statistically significant difference between the concentration of reversine and the gene expression of Oct4 ( *p* = 0.004), with the greatest Oct4 gene expression in the control group and the lowest Oct4 gene expression in the REV40 group, as can be seen in Figure 2.

**Figure 3.**
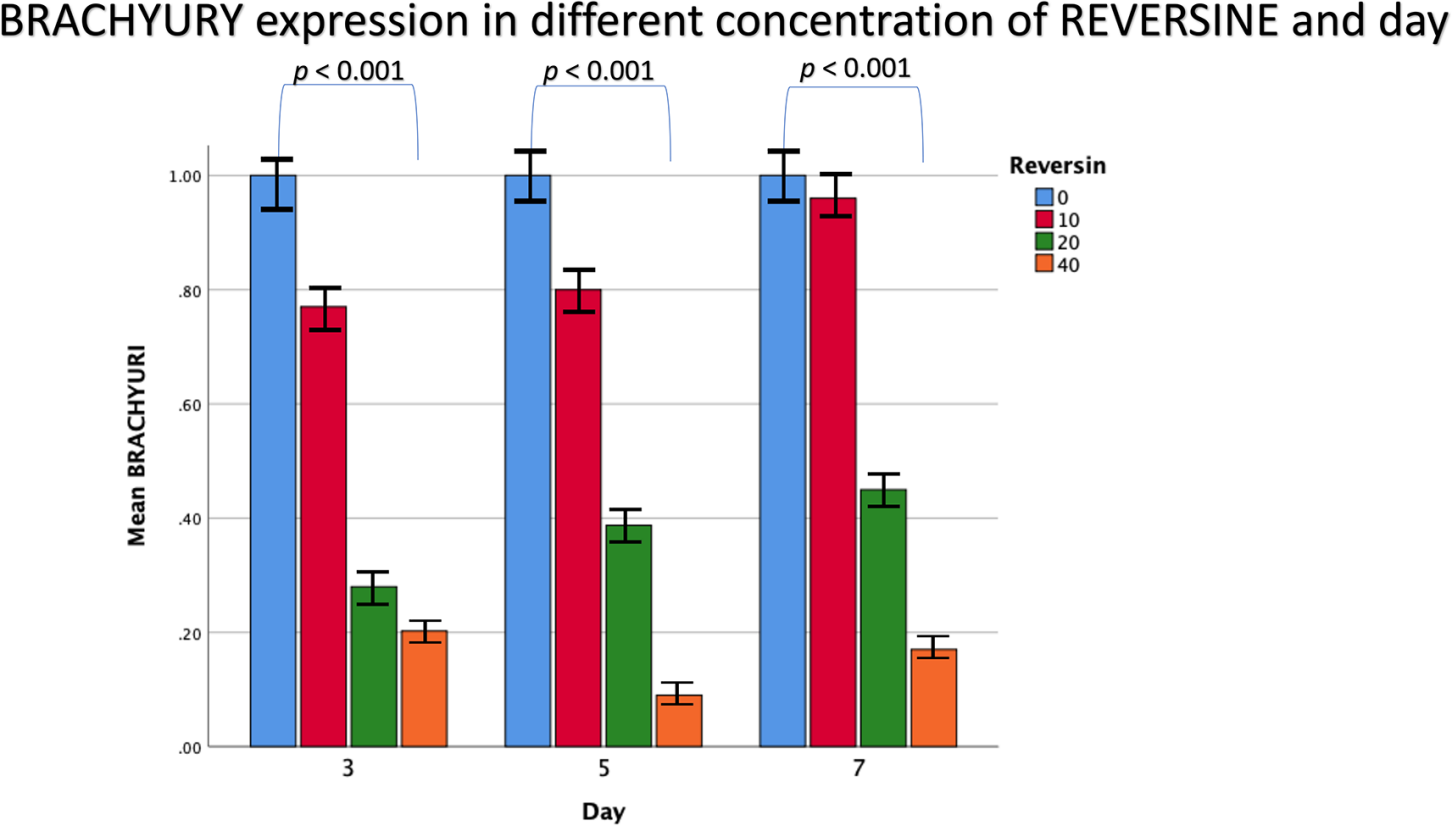
Graph showing that brachyury gene expression is highest in the control group, and decreased in the REV10, REV20 and REV40 groups. REV, reversine.

The mean value for brachyury expression from the 6 ^th^ cell passage showed a decreasing trend with the addition of reversine. There was a statistically significant difference between the concentration of reversine and the gene expression of brachyury ( *p* < 0.001), with the greatest brachyury gene expression in the control group and the lowest brachyury gene expression in the REV40 group, as can be seen in Figure 3.

**Figure 4.**
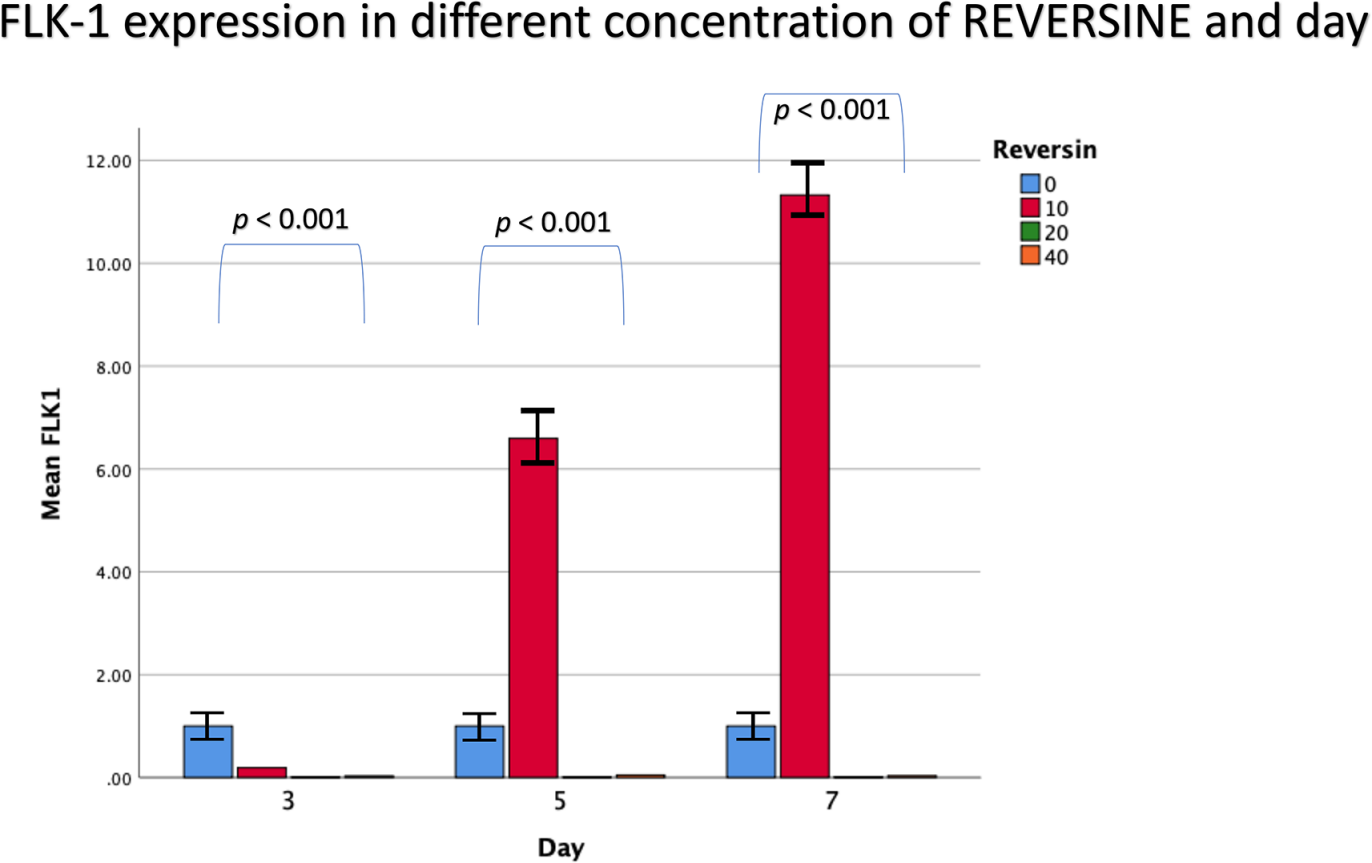
Graph showing that Flk-1 gene expression is highest in the REV10 group compared with the control, REV20 and REV40 groups. Flk-1, Fetal liver kinase 1; REV, reversine.

For Flk-1 gene expression from the 6 ^th^ cell passage, REV10 increased the mean value of Flk-1 expression from the 3 ^rd^ day of observation until the 7 ^th^ day of observation, whereas REV20 and REV40 did not show any statistically significant differences ( Figure 4).

**Figure 5.**
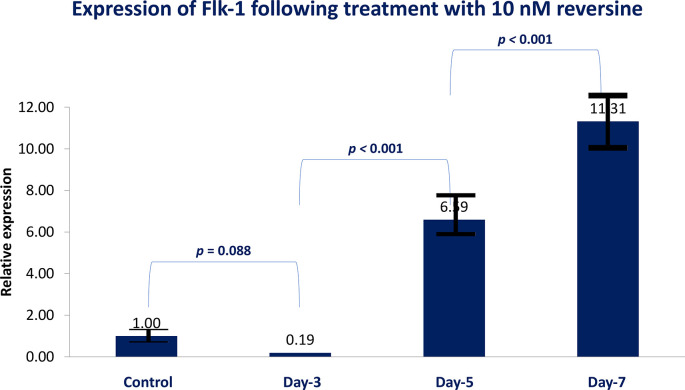
Diagram showing the expression of Flk-1 following treatment with 10 nM reversine based on daily trend when compared with the control group. Flk-1, Fetal liver kinase 1.

If the REV20 and REV40 variables are omitted, it can be concluded that REV10 significantly increased the mean value of Flk-1 gene expression on the 5 ^th^ and 7 ^th^ day of observation ( *p* < 0.001). These data were obtained through analysis using independent t-test and paired t-test, with a bar chart in Figure 5.

### Immunofluorescence analysis of cardiomyocyte cell markers

**Figure 6.**
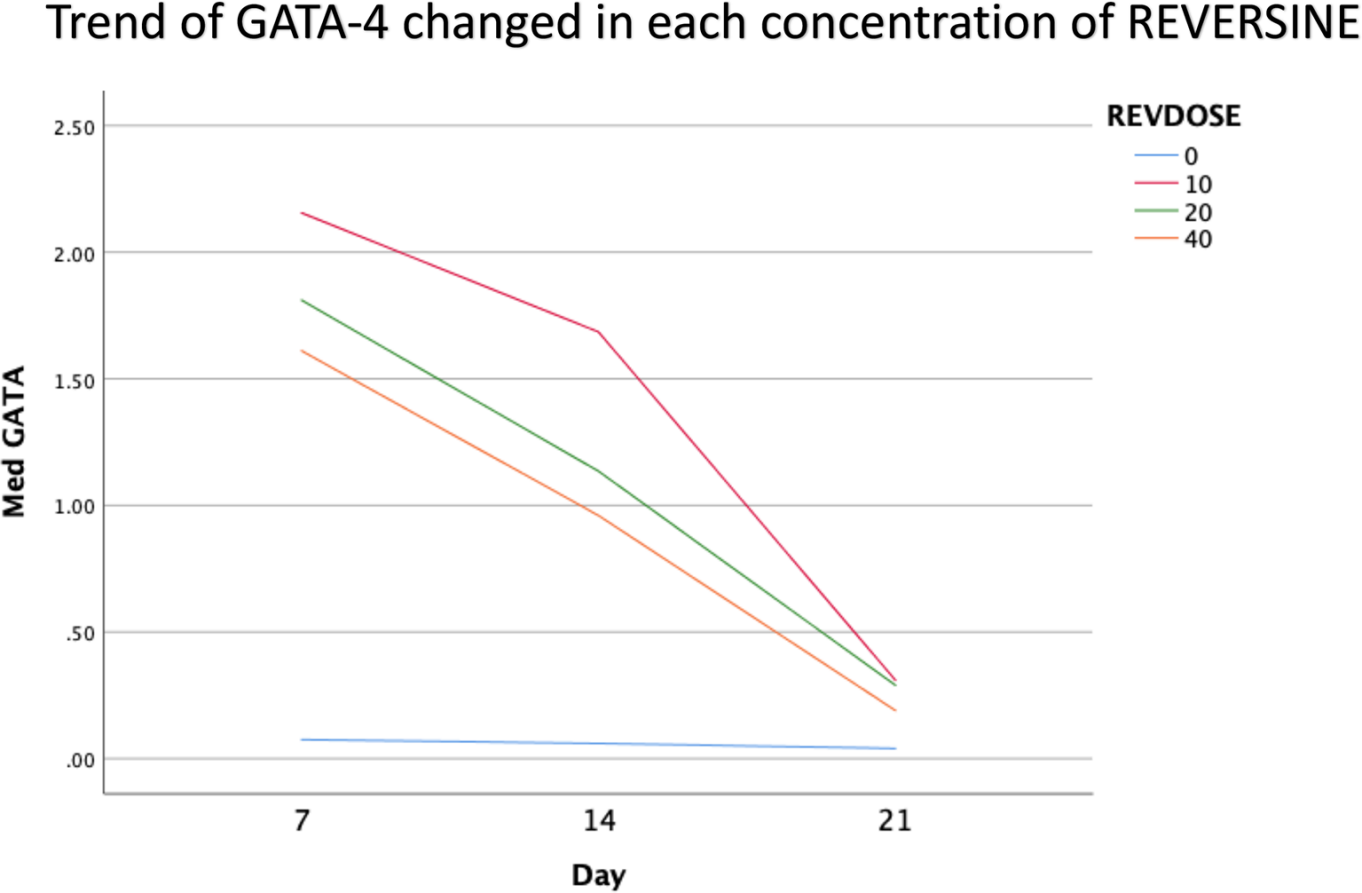
Graph showing the decreasing expression of GATA4 in all groups over 21 days of observation. GATA4, Transcription factor GATA-4; REV, reversine.

**Figure 7.**
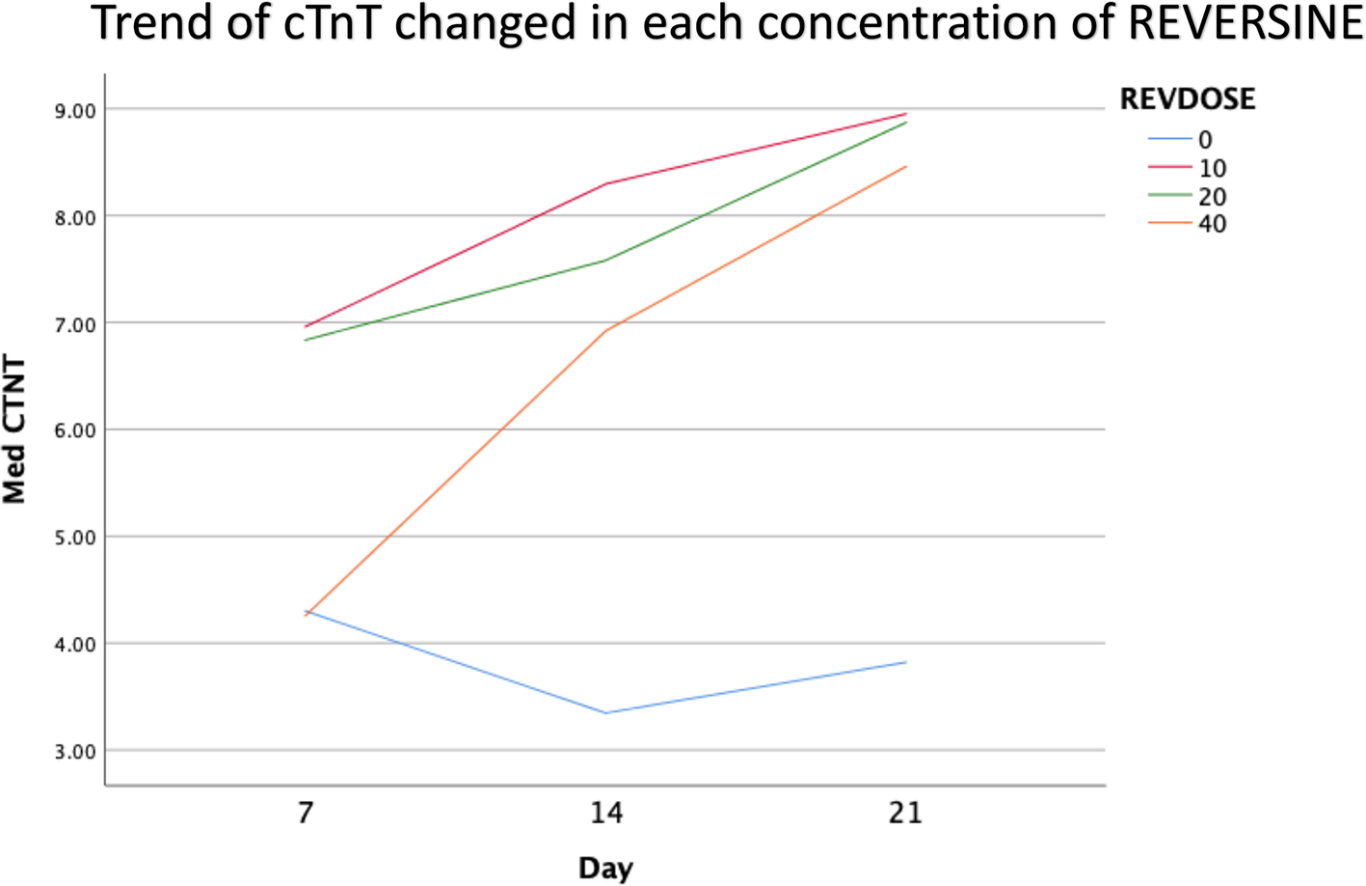
Graph showing the increasing expression of cTnT in all groups over 21 days of observation. cTnT, Cardiac troponin T; REV, reversine.

The quantity of GATA-4 and cTnT expression from the 6 ^th^ cell passage analysed on day 21 showed an abnormal data distribution, so we used the Kruskal-Wallis test for further data processing. The median value of GATA-4 decreased from day 7 until day 21, while the median value of cTnT increased from day 7 until day 21. Kruskal-Wallis test revealed that the expression levels of GATA-4 and cTnT were different between control group, REV10, REV20 and REV40 groups ( *p* < 0.001) as can be seen in Table 3. Figure 6 revealed a trend of decreasing value of GATA4 in all groups between 21 days of observation ( *p* = 0.017). Figure 7 revealed a trend of increasing value of cTnT in all group between 21 days of observation ( *p* = 0.001).

**Table 3.** Median value and Kruskal-Wallis test for GATA4 and cTnT based on the concentration of reversine.

Variable	REV dose	Median rank	Kruskal-Wallis	Sig
GATAD7	0	4.50	21.830	.000
	1	25.75		
	2	19.69		
	3	16.06		
GATAD14	0	4.63	20.091	.000
	1	25.13		
	2	18.25		
	3	18.00		
GATAD21	0	4.69	19.908	.000
	1	23.75		
	2	20.69		
	3	16.88		
CTNTD7	0	10.13	13.526	.000
	1	24.00		
	2	21.00		
	3	10.88		
CTNTD14	0	6.75	16.787	.000
	1	25.00		
	2	20.00		
	3	14.25		
CTNTD21	0	8.38	8.483	.000
	1	20.88		
	2	19.13		
	3	17.63		

GATA4, Transcription factor GATA-4; cTnT, Cardiac troponin T; REV, reversine.

### Path analysis

**Figure 8.**
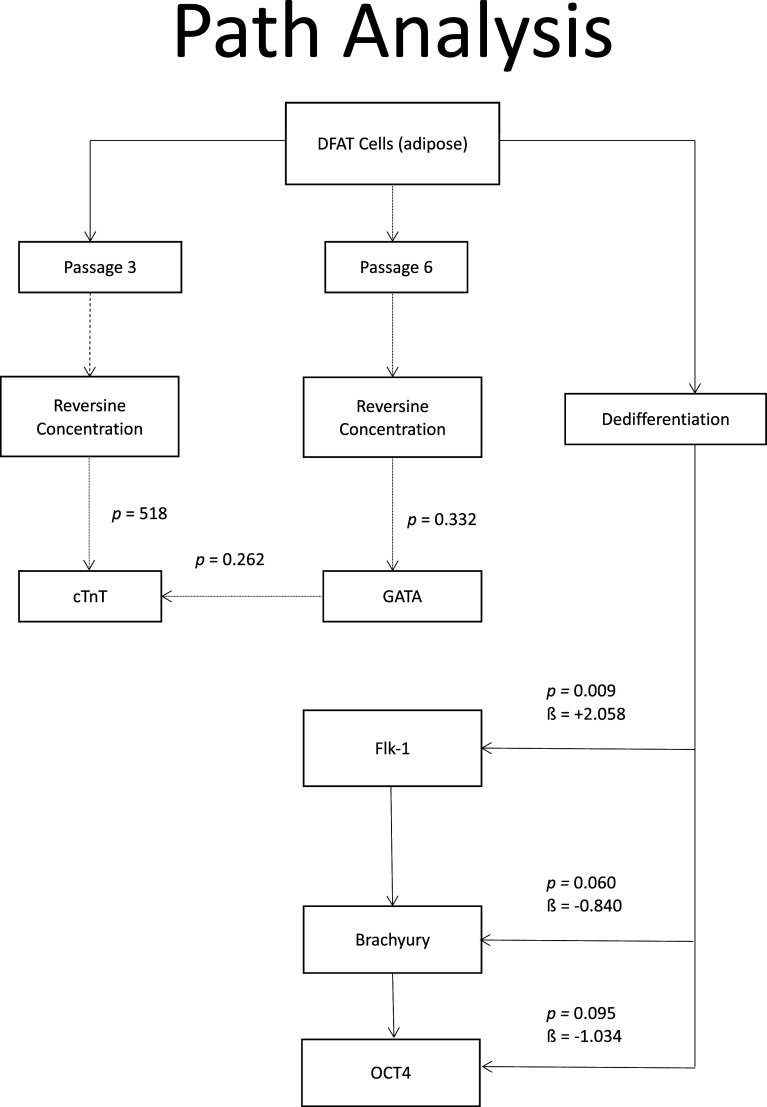
Path analysis for relationship between variables of reversine concentration on differentiation of DFAT cells into cardiomyocytes. Oct4, Octamer-binding transcription factor 4; Flk-1, Fetal liver kinase 1; GATA4, Transcription factor GATA-4; cTnT, Cardiac troponin T.

When path analysis was performed on all variables, it was found that at phase 3, the dose of reversine had a significant effect on the quantity of cTnT ( *p* = 0.015) and GATA4 ( *p* = 0.011) expression. Meanwhile, in passage six, the reversine dose only had a significant effect on GATA4 expression ( *p* = 0.004). Meanwhile, the reversine dose had a significant effect on the quantity of Flk-1 expression ( *p* < 0.001), which indicates how strong the effect of the dose is (a positive sign means it has a positive effect as can be seen in Figure 8.

**Figure 9.**
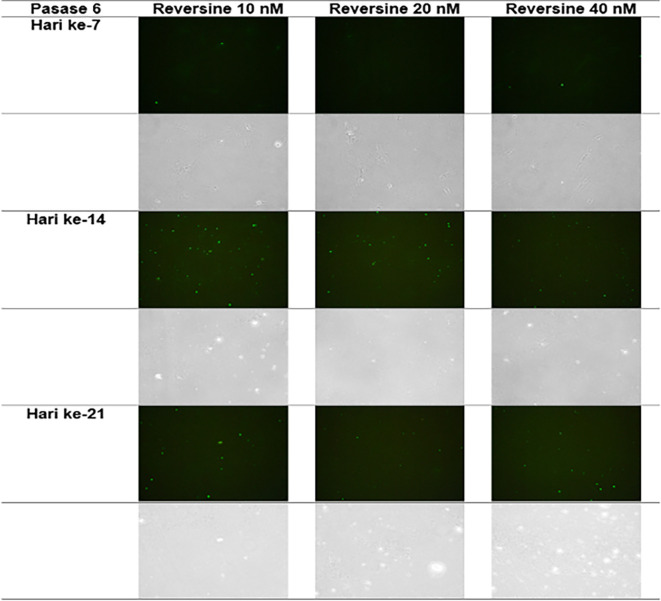
Immunofluorescence of GATA4 in the cell culture.

**Figure 10.**
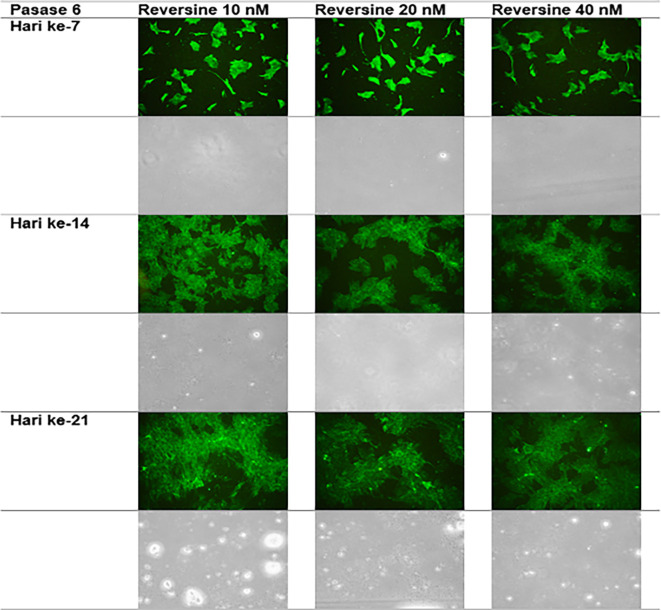
Immunofluorescence of cTnT in the cell culture.

## Discussion

### Expression of cell potency and DFAT “cell stemness”

The ability of DFAT cells to differentiate into multiple derived cells from the three germ layers raises questions about the cell stemness properties of DFAT cells. Previous studies have shown that DFAT cells have similar properties to mesenchymal stem cells. ^25^^–^^30^ DFAT cells show the expression of several cell potency such as Oct4, Sox2, Nanog, Rex-1 ^31^ SOX9, ACAN ^32^ and Flk-1. ^33^ In this study, DFAT cells were exposed to different doses of reversine with the assumption that the reversine would affect the potency of DFAT cells. There were three markers analysed in this study, namely Oct4, which represents cell stemness transcription factor (SRTF) at the pluripotent level, brachyury, which represents mesodermal cell transcription factor at the multipotent level, and Flk-1, which is a marker of mesodermal endothelial cells. Meanwhile, the doses of reversine used were 10, 20 and 40 nM. The concentration selection was made based on a preliminary study that had been carried out previously using reversine at the concentration of 10, 20 and 40 nM. Analysis of these three markers showed that reversine, especially at the concentration of 10 nM, was able to increase Flk-1 expression consistently during the three days of observation. Reversine at the concentration of 10 nM was also superior to other doses in increasing the expression of Oct4 and brachyury markers even though it had a lower mean value than the control group in all observation groups.

### Expression of Flk-1 as a marker of mesodermal endothelial cells

Flk-1 is one of the markers of mesodermal endothelial cells that are essential in the development of endothelial and other hematopoietic derivatives. The detection of this marker confirms the assumption that DFAT cells undergo dedifferentiation from mature mesenchymal cells into more “younger” cells. Expression of Flk-1 in DFAT cells indicates the potency of these cells to differentiate into cardiomyocytes. This is in line with previous studies using an embryonic stem cell differentiation model in mice, showing that progenitor cells expressing Flk-1 can differentiate into contractile cardiomyocytes, whereas cells that do not express Flk-1 are unable to differentiate into contractile cardiomyocytes.

### Expression of brachyury as a carcinoma cell transcription factor

Brachyury is a transcription factor that indicates the ability of mesodermal differentiation. Brachyury expression in DFAT cells notably decreases when compared to AMSC cells. ^34^ In this study, the administration of reversine did not increase the expression of brachyury. Perhaps this indicates the ability of reversine to induce further de-differentiation in DFAT cells not to the mesodermal level. Brachyury is also a specific and sensitive marker for the diagnosis of chordoma and has been shown to play a role in the carcinogenesis and development of several epithelial carcinomas. ^35^ A further question that needs to be investigated is whether the low expression of brachyury is associated with a lower risk of DFAT cell carcinogenesis. ^36^

### Oct4 expression as a marker of cell pluripotency

Oct4 is one of the markers of cell pluripotency that plays a role in the differentiation of the three germ layers. Oct4 expression in this study is lower than in several previous studies. ^35^ This could be due to several reasons, the first being the lack of observation days and the second being that the dose in this study was not able to induce an increase in Oct4 expression.

### Effect of reversine concentration to cTnT and GATA4 expression

One of the main objectives in this study was to find out the optimal dose for differentiation of DFAT cells into cardiomyocytes. The results of this study indicate that reversine had a significant effect on the expression of cardiomyocyte cell markers. Among the three doses used, reversine at a dose of 10 nM was consistently able to increase the expression of GATA4 and cTnT markers in two different passage groups. Meanwhile, the other two doses, namely the concentration of 20 nM and 40 nM, showed inferior results when compared with the concentration of 10 nM. This supports the assumption that the dose of reversine is concentration dependent on the differentiation ability of DFAT cells.

If you look at the median values of GATA4 and cTnT in each subgroup, you will see a tendency to decrease the median value of GATA4 and increase the median value of cTnT on each day of observation. This is consistent with the theory that states that GATA4 as a marker of cardiac progenitor cells will decrease when DFAT cells differentiate into cardiomyocytes, which are characterized by the detection of cTnT, so it is assumed that GATA4 and cTnT have a negative association, *i.e.*, the lower GATA4 expression the higher the levels of cTnT. However, a different reality was found in this study, particularly in the cTnT group. In the GATA4 group, all concentrations of reversine consistently showed a lower median value as the days of observation increased, with reversine at a concentration of 10 nM having the largest effect on decreasing GATA4 values in culture cells. ^37^^–^^39^

In the cTnT group, inconsistency was seen in each passage group, reversine at a concentration of 10 nM increased the median value on days 7 and 14, but decreased values on day 21 in passage three, while in passage six the median value consistently increased. The same thing happened in the 40 nM reversine group. Meanwhile, at a concentration of 20 nM, the median value at passages three and six seemed to decrease with increasing days of observation.

### Limitations

The inconsistencies mentioned above can be caused by several technical factors that were not controlled by the researchers, including cell density, which causes false negative results, the emergence of artifacts during staining, which causes false positive results and less than perfect absorption of antibody/Alexa Fluor due to insufficient membrane recovery time achieved. Although passages three and six are still classified as early passages, there are several things that can cause differences in the quality of each of these passages. Senescence is one of the causes of decreased differentiation ability of DFAT cells. It can be concluded that the differentiation ability decreases as cells age.

## Conclusions

Reversine could increase the expression of Flk-1, yet it was unable to stimulate the expression of Oct4 and brachyury related to cell stemness. An optimal concentration of 10 nM reversine may have the greatest effect on enhancing the differentiation of DFAT cells into mature cardiomyocytes, as can be seen by higher cTnT expression between cells.

## Data availability

### Underlying data

Figshare: Raw Data - Effects of Different Concentration of Reversine to Enhance Conversion of Dedifferentiated Fat Cells into Cardiomyocyte. https://doi.org/10.6084/m9.figshare.20000426. ^22^

The project contains the following underlying data:

• ICC-BB.sav (immunocytochemistry data)
• RTqPCR.sav

Figshare: Raw Ct values and standard curves for all samples and replicates. https://doi.org/10.6084/m9.figshare.20109680. ^23^

Figshare: Raw, unedited, uncropped immunocytochemistry images. https://doi.org/10.6084/m9.figshare.20109629. ^24^

Data are available under the terms of the Creative Commons Attribution 4.0 International license (CC-BY 4.0).

## References

[1] Sampogna G Guraya SY Forgione A : Regenerative medicine: Historical roots and potential strategies in modern medicine. *J. Microsc. Ultrastruct.* 2015;3:101–107. 10.1016/j.jmau.2015.05.002 30023189 PMC6014277

[2] Duarte MS Bueno R Silva W : Triennial growth and development symposium: Dedifferentiated fat cells: Potential and perspectives for their use in clinical and animal science purpose. *J. Anim. Sci.* 2017;95(5):2255–2260. 10.2527/jas2016.1094 28727019

[3] Dufrane D : Impact of Age on Human Adipose Stem Cells for Bone Tissue Engineering. *Cell Transplant.* 2017;26:1496–1504. 10.1177/0963689717721203 29113460 PMC5680951

[4] Fitzsimmons REB Mazurek MS Soos A : Mesenchymal stromal/stem cells in regenerative medicine and tissue engineering. *Stem Cells Int.* 2018;2018:1–16. 10.1155/2018/8031718 30210552 PMC6120267

[5] Gao Q Zhao L Song Z : Expression pattern of embryonic stem cell markers in DFAT cells and ADSCs. *Mol. Biol. Rep.* 2012;39(5):5791–5804. 10.1007/s11033-011-1371-4 22237862

[6] Giordano A Frontini A Cinti S : Convertible visceral fat as a therapeutic target to curb obesity. *Nat. Rev. Drug Discov.* 2016;15(6):405–424. 10.1038/nrd.2016.31 26965204

[7] Mitrečić D Hribljan V Jagečić D : Regenerative Neurology and Regenerative Cardiology: Shared Hurdles and Achievements. *Int. J. Mol. Sci.* 2022;23(2):855. 10.3390/ijms23020855 35055039 PMC8776151

[8] Gu Y Li T Ding Y : Changes in mesenchymal stem cells following long-term culture in vitro. *Mol. Med. Rep.* 2016;13(6):5207–5215. 10.3892/mmr.2016.5169 27108540

[9] Hong SH Lee MH Koo MA : Stem cell passage affects directional migration of stem cells in electrotaxis. *Stem Cell Res.* 2019;38(January):101475. 10.1016/j.scr.2019.101475 31176110

[10] Huang Y Huang D Weng J : Effect of reversine on cell cycle, apoptosis, and activation of hepatic stellate cells. *Mol. Cell. Biochem.* 2016;423(1–2):9–20. 10.1007/s11010-016-2815-x 27734224

[11] Jeziorowska D Korniat A Salem JE : Generating patient-specific induced pluripotent stem cells-derived cardiomyocytes for the treatment of cardiac diseases. *Expert. Opin. Biol. Ther.* 2015;15(10):1399–1409. 10.1517/14712598.2015.1064109 26134098

[12] Kikuta S Tanaka N Kazama T : Osteogenic Effects of Dedifferentiated Fat Cell Transplantation in Rabbit Models of Bone Defect and Ovariectomy-Induced Osteoporosis. *Tissue Eng. A.* 2013;19(15–16):1792–1802. 10.1089/ten.tea.2012.0380 23566022 PMC3700015

[13] Li XC Guo Y Yao Y : Reversine increases the plasticity of long-term cryopreserved fibroblasts to multipotent progenitor cells through activation of Oct4. *Int. J. Biol. Sci.* 2016;12(1):53–62. 10.7150/ijbs.12199 26722217 PMC4679398

[14] Liu M Lei H Dong P : Adipose-Derived Mesenchymal Stem Cells from the Elderly Exhibit Decreased Migration and Differentiation Abilities with Senescent Properties. *Cell Transplant.* 2017;26:1505–1519. 10.1177/0963689717721221 29113467 PMC5680952

[15] McGown C Birerdinc A Younossi ZM : Adipose tissue as an endocrine organ. *Clin. Liver Dis.* 2014;18(1):41–58. 10.1016/j.cld.2013.09.012 24274864

[16] Poloni A Maurizi G Leoni P : Human dedifferentiated adipocytes show similar properties to bone marrow-derived mesenchymal stem cells. *Stem Cells.* 2012;30(5):965–974. 10.1002/stem.1067 22367678

[17] Qu G Schroeder HP von : Preliminary Evidence for the Dedifferentiation of RAW 264.7 Cells into Mesenchymal Progenitor-Like Cells by a Purine Analog. *Tissue Eng. A.* 2012;18:1890–1901. 10.1089/ten.tea.2010.0692 22519969

[18] Santaguida S Tighe A D’Alise AM : Dissecting the role of MPS1 in chromosome biorientation and the spindle checkpoint through the small molecule inhibitor reversine. *J. Cell Biol.* 2010;190:73–87. 10.1083/jcb.201001036 20624901 PMC2911657

[19] Saraiya M Nasser R Zeng Y : Reversine enhances generation of progenitor-like cells by dedifferentiation of annulus fibrosus cells. *Tissue Eng. Part A.* 2010;16:1443–1455. 10.1089/ten.tea.2009.0343 19947906 PMC2952128

[20] Berthouex PM Brown LC : *Statistics for Environmental Engineers.* 2nd ed. Boca Raton, FL: Lewis Publishers;2002.

[21] Livak KJ Schmittgen TD : Analysis of relative gene expression data using real-time quantitative PCR and the 2(-Delta C(T)) Method. *Methods.* 2001 Dec;25(4):402–408. 10.1006/meth.2001.1262 11846609

[22] Nugraha RA Baktijasa B Sargowo D : Raw Data - Effects of Different Concentration of Reversine to Enhance Conversion of Dedifferentiated Fat Cells into Cardiomyocyte. figshare. [Dataset]. 2022. 10.6084/m9.figshare.20000426.v1

[23] Nugraha RA Baktijasa B Sargowo D : Raw Ct values and standard curves for all samples and replicates. figshare. Dataset. 2022. 10.6084/m9.figshare.20109680.v1

[24] Nugraha RA Baktijasa B Sargowo D : Raw, unedited, uncropped immunocytochemistry images. figshare. Figure. 2022. 10.6084/m9.figshare.20109629.v1

[25] Shah SR David JM Tippens ND : Brachyury-YAP Regulatory Axis Drives Stemness and Growth in Cancer. *Cell Rep.* 2017;21(2):495–507. 10.1016/j.celrep.2017.09.057 29020634 PMC5637538

[26] Shall G Menosky M Decker S : Effects of Passage Number and Differentiation Protocol on the Generation of Dopaminergic Neurons from Rat Bone Marrow-Derived Mesenchymal Stem Cells. *Int. J. Mol. Sci.* 2018;19(3):1–31. 10.3390/ijms19030720 29498713 PMC5877581

[27] Soltani L Rahmani HR Daliri Joupari M : Effects of Different Concentrations of Reversine on Plasticity of Mesenchymal Stem Cells. *Ind. J. Clin. Biochem.* December 2018;35:188–196. 10.1007/s12291-018-0800-8 32226250 PMC7093655

[28] Schubert C von Cubizolles F Bracher JM : Plk1 and Mps1 Cooperatively Regulate the Spindle Assembly Checkpoint in Human Cells. *Cell Rep.* 2015;12(1):66–78. 10.1016/j.celrep.2015.06.007 26119734

[29] Wei S Duarte MS Zan L : Cellular and Molecular Implications of Mature Adipocyte Dedifferentiation. *J. Genomics.* 2013;1:5–12. 10.7150/jgen.3769 25031650 PMC4091435

[30] Ziaeian B Fonarow GC : Epidemiology and aetiology of heart failure. *Nat. Rev. Cardiol.* 2016;13(6):368–378. 10.1038/nrcardio.2016.25 26935038 PMC4868779

[31] Zuk PA Zhu M Ashjian P : Human Adipose Tissue Is a Source of Multipotent Stem Cells. *Mol. Biol. Cell.* 2002;13(12):4279–4295. 10.1091/mbc.e02-02-0105 12475952 PMC138633

[32] Saler M Caliogna L Botta L : hASC and DFAT, multipotent stem cells for regenerative medicine: A comparison of their potential differentiation in vitro. *Int. J. Mol. Sci.* 2017;18. 10.3390/ijms18122699 29236047 PMC5751300

[33] Jumabay M Zhang R Yao Y : Spontaneously beating cardiomyocytes derived from white mature adipocytes. *Cardiovasc. Res.* 2010;85(1):17–27. 10.1093/cvr/cvp267 19643806 PMC2791054

[34] Jumabay M Abdmaulen R Urs S : Endothelial differentiation in multipotent cells derived from mouse and human white mature adipocytes. *J. Mol. Cell. Cardiol.* 2012;53:790–800. 10.1016/j.yjmcc.2012.09.005 22999861 PMC3523675

[35] Figiel-dabrowska A Radoszkiewicz K Rybkowska P : Neurogenic and neuroprotective potential of stem/stromal cells derived from adipose tissue. *Cells.* 2021;10(6):1–27. 10.3390/cells10061475 34208414 PMC8231154

[36] Chen M Wu Y Zhang H : The Roles of Embryonic Transcription Factor BRACHYURY in Tumorigenesis and Progression. *Front. Oncol.* 2020;10(June):1–9. 10.3389/fonc.2020.00961 32695672 PMC7338565

[37] Badimon L Oñate B Vilahur G : Adipose-derived mesenchymal stem cells and their reparative potential in ischemic heart disease. *Rev. Esp. Cardiol.* 2015;68:599–611. 10.1016/j.recesp.2015.02.025 26028258

[38] Chen L Qin F Ge M : Application of Adipose-Derived Stem Cells in Heart Disease. *J. Cardiovasc. Transl. Res.* 2014;7(7):651–663. 10.1007/s12265-014-9585-1 25205213

[39] Jumabay M : Dedifferentiated fat cells: A cell source for regenerative medicine. *World J. Stem Cells.* 2015;7:1202–1214. 10.4252/wjsc.v7.i10.1202 26640620 PMC4663373

