# Supplementary material for: Reversine enhances the conversion of dedifferentiated fat cells into mature cardiomyocytes
Source: F1000Res. 2024 Jun 11;11:851. Originally published 2022 Jul 28. [Version 3] doi: 10.12688/f1000research.122788.3 (PMC11270644; doi:10.12688/f1000research.122788.3)
Supplement: Peer review history

# Reviewer report: Reviewer response for version 3

**DOI:** 10.5256/f1000research.167666.r289885

**Published:** 2024-07-23

**Authors:** Jelena Milasin

**Affiliations:** 1 Department of Human Genetics, School of Dental Medicine, University of Belgrade, Belgrade, Serbia

The authors have addressed in a satisfactory way most of the issues raised by this reviewer and thus the paper has been considerably improved.

Is the work clearly and accurately presented and does it cite the current literature?

Partly

If applicable, is the statistical analysis and its interpretation appropriate?

Yes

Are all the source data underlying the results available to ensure full reproducibility?

Partly

Is the study design appropriate and is the work technically sound?

Partly

Are the conclusions drawn adequately supported by the results?

Partly

Are sufficient details of methods and analysis provided to allow replication by others?

Partly

Reviewer Expertise:

stem cell studies (characterization, differentiation).

I confirm that I have read this submission and believe that I have an appropriate level of expertise to confirm that it is of an acceptable scientific standard.

# Reviewer report: Reviewer response for version 2

**DOI:** 10.5256/f1000research.163823.r270562

**Published:** 2024-06-06

**Authors:** Jelena Milasin

**Affiliations:** 1 Department of Human Genetics, School of Dental Medicine, University of Belgrade, Belgrade, Serbia

The topic of the paper „Effects of different concentrations of reversine to enhance conversion of dedifferentiated fat cells into mature cardiomyocytes“, is interesting and relevant to the field of regenerative medicine. The authors have found that reversine, a purine-derivative, could stimulate the differentiation of DFAT cells into mature cardiomyocytes, as indicated by the higher expression of cardiac troponin T, a specific marker of cardiac muscle cells.

 Although the study is interesting, it has many imprecisions and linguistic flaws leading to ambiguities.

**Title:**

 Rephrase slightly („Effects of different concentrations of reversine on the conversion of dedifferentiated fat cells into mature cardiomyocytes“).

**Introduction:**

- Authors should cite the review article ( Mitrečić D.et.al., 2022 [Ref 1])
- Authors must  rephrase the following sentence as it is unclear: “... a higher percentage of the expressed quantity of the mesenchymal stem cell marker known as dedifferentiated fat (DFAT) cells”. It should be either “higher quantity” or “higher percentage” of expressed markers. And markers ARE NOT dedifferentiated cells.
- Delete “more” in the sentence “...the dedifferentiation of unipotent myoblast cells to progenitor cells, which are **more **multipotent”. In the next sentence delete “stem”. The sentence should read: „Reversine as an inductor of cell dedifferentiation...”
- “Aims” and “objectives” represent a redundancy. The two paragraphs should be merged, re-worded and shortened.

**M&M:**

- It is unclear what was the number of patients whose adipose tissues were used for cell culturing.
- All units should be very carefully re-checked. For instance „A total of 30-50 L was then transferred” or “A total of 2 L reagent was added to the PCR tube, then 3 L RNA sample was added to the same PCR tube”- **µ** is missing; also “5.6 microns carrier RNA and 560 micros AVL buffer”-this is incorrect, etc.
- Additionally, authors say 30-50 microliters was transferred into 6-well plates. It changes a lot if there was 20 microliters in one and 50 microliters in another well. Correct this.
- What was done with DFAT after 15 minutes of incubation with reversine?

**Results:**

- Under the subsection “Analysis of RT-qPCR for potential markers of DFAT”, there are some discrepancies. Authors say that Oct and Brachyury had lower average values than controls, and that for Flk it was the opposite, when even Flk had lower values compared to controls. Either correct this statement or the table.
- Maybe authors should merge this and the following subsection (as it also deals with relative expressions of Oct, brachyury and Flk genes) and harmonize them.
- The word “gene” is missing after Oct, brachyury, Flk (it should be Oct4 gene expression, etc.)
- The standard deviation bars are missing on the histograms
- It is unclear which cell passage (3 ^rd^ or 6 ^th^) treatments are given in the figures.
- It is not very clear what do the *p* values above the histogram actually connect.

Figure 2. caption should be corrected (“Graph showing that Oct4 expression is highest in the control group, and decreased in the REV10, REV20 and REV40 groups”), because for REV10, on day 7, there is an increase.

**Discussion:**

 Rather use the word “potency” instead of potential in this case, and correct “stem cell-ness” into “cell stemness”.

 The expression of Oct in this paper and the paper #34 is not really comparable, because the experimental settings are different.

 English language needs improvements.

Is the work clearly and accurately presented and does it cite the current literature?

Partly

If applicable, is the statistical analysis and its interpretation appropriate?

Yes

Are all the source data underlying the results available to ensure full reproducibility?

Partly

Is the study design appropriate and is the work technically sound?

Partly

Are the conclusions drawn adequately supported by the results?

Partly

Are sufficient details of methods and analysis provided to allow replication by others?

Partly

Reviewer Expertise:

stem cell studies (characterization, differentiation).

I confirm that I have read this submission and believe that I have an appropriate level of expertise to confirm that it is of an acceptable scientific standard, however I have significant reservations, as outlined above.

## References

[1] : Regenerative Neurology and Regenerative Cardiology: Shared Hurdles and Achievements. *Int J Mol Sci* .2022;23(2) : 10.3390/ijms23020855 10.3390/ijms23020855 35055039 PMC8776151

# Response

**Published:** 2024-06-07

**Authors:** Ricardo Adrian Nugraha

**Affiliations:** Airlangga University, Indonesia

The topic of the paper „Effects of different concentrations of reversine to enhance conversion of dedifferentiated fat cells into mature cardiomyocytes“, is interesting and relevant to the field of regenerative medicine. The authors have found that reversine, a purine-derivative, could stimulate the differentiation of DFAT cells into mature cardiomyocytes, as indicated by the higher expression of cardiac troponin T, a specific marker of cardiac muscle cells.

 Although the study is interesting, it has many imprecisions and linguistic flaws leading to ambiguities.

**Title:**

 Rephrase slightly („Effects of different concentrations of reversine on the conversion of dedifferentiated fat cells into mature cardiomyocytes“).

 Author response: thank you for your suggestion. We rephrased the title with “Reversine enhances the conversion of dedifferentiated fat cells into mature cardiomyocytes”

**Introduction:**

- Authors should cite the review article ( Mitrečić D.et.al., 2022 [Ref 1])

Author response: thank you for your suggestion. We cited the article you’ve suggested in the reference number 7.

- Authors must  rephrase the following sentence as it is unclear: “... a higher percentage of the expressed quantity of the mesenchymal stem cell marker known as dedifferentiated fat (DFAT) cells”. It should be either “higher quantity” or “higher percentage” of expressed markers. And markers ARE NOT dedifferentiated cells.

Author response: thank you for your feedback. We rephrased it with “… with a higher quantity of dedifferentiated fat (DFAT) cells”

- Delete “more” in the sentence “...the dedifferentiation of unipotent myoblast cells to progenitor cells, which are **more **multipotent”. In the next sentence delete “stem”. The sentence should read: „Reversine as an inductor of cell dedifferentiation...”

Author response: thank you for your feedback. We deleted “more” and “stem” as you have suggested.

- “Aims” and “objectives” represent a redundancy. The two paragraphs should be merged, re-worded and shortened.

Author response: thank you for your suggestion. We merged and shortened these two paragraphs into one.

**M&M:**

- It is unclear what was the number of patients whose adipose tissues were used for cell culturing.

Author response: we got it from one patient, a 39-years-old woman who underwent surgery for hernia.

- All units should be very carefully re-checked. For instance „A total of 30-50 L was then transferred” or “A total of 2 L reagent was added to the PCR tube, then 3 L RNA sample was added to the same PCR tube”- **µ** is missing; also “5.6 microns carrier RNA and 560 micros AVL buffer”-this is incorrect, etc.

Author response: thank you for your very careful observation. We apologized for several missing words and errors. We have fixed it.

- Additionally, authors say 30-50 microliters was transferred into 6-well plates. It changes a lot if there was 20 microliters in one and 50 microliters in another well. Correct this.

Author response: thank you for your very careful observation. Actually we used 30 µL. We have fixed it. We apologize for this error.

- What was done with DFAT after 15 minutes of incubation with reversine?

Author response: The cells were pretreated with 10, 20, and 40 nM of reversine for 15 minutes, and then further induced under lineage-specific inducing condition in differentiation medium. Cells grown on coverslips were fixed with 4% paraformaldehyde. Then, we performed RT-qPCR for Oct4, brachyury and Flk-1 on days 7, 14 and 21 after the cells were exposed to differentiation medium. We performed quantification of dedifferentiating mature adipocytes and cardiomyocyte expression (GATA4 and cTnT) were performed by immunocytochemistry.

**Results:**

- Under the subsection “Analysis of RT-qPCR for potential markers of DFAT”, there are some discrepancies. Authors say that Oct and Brachyury had lower average values than controls, and that for Flk it was the opposite, when even Flk had lower values compared to controls. Either correct this statement or the table.

Author response: thank you for your careful observation and feedback. We apologize for the misunderstanding. We have corrected the table. The true statemet is reversine groups always have higher mean value of Flk than control group, with REV40 had the highest value of Flk-1.

- Maybe authors should merge this and the following subsection (as it also deals with relative expressions of Oct, brachyury and Flk genes) and harmonize them.

Author response: thank you for your suggestion, we merged both and deleted the following subsections.

- The word “gene” is missing after Oct, brachyury, Flk (it should be Oct4 gene expression, etc.)

Author response: thank you for your suggestion, we added “gene expression”

- The standard deviation bars are missing on the histograms

Author response: thank you for your consideration and attention to this matter.

 We added the standard deviation bars on the histograms.

- It is unclear which cell passage (3 ^rd^or 6 ^th^) treatments are given in the figures.

Author response: we used the 6 ^th^ cell passage treatments for data analysis and figures. We will explain it in the manuscript.

- It is not very clear what do the *p *values above the histogram actually connect.

Author response: thank you for your consideration and attention to this matter. We try to explain the *p values *clearer, as a comparison between each group of intervention, not between day *.*

 Figure 2. caption should be corrected (“Graph showing that Oct4 expression is highest in the control group, and decreased in the REV10, REV20 and REV40 groups”), because for REV10, on day 7, there is an increase.

 Author response: thank you for your suggestion, we have corrected the Figure 2 caption with “except on day 7”.

**Discussion:**

 Rather use the word “potency” instead of potential in this case, and correct “stem cell-ness” into “cell stemness”.

 Author response: thank you for your suggestion, we replace the word “potential” with “potency” and “stem cell-ness” with “cell stemness”

 The expression of Oct in this paper and the paper #34 is not really comparable, because the experimental settings are different.

 Author response: we agreed with you, since there are several differences in the experimental setting, thus it couldn’t be compared.

 English language needs improvements.

 Author response: thank you for your suggestion. We have sent it to the proof language service to improve our grammars.

# Reviewer report: Reviewer response for version 2

**DOI:** 10.5256/f1000research.163823.r258434

**Published:** 2024-04-09

**Authors:** Francesco Galli

**Affiliations:** 1 The University of Manchester, Manchester, England, UK

The authors addressed the primary request from the initial review by including Immunofluorescence images of OCT4 and cTnT. Additionally, they enhanced the clarity and strength of the manuscript by improving the explanation of the results.

Is the work clearly and accurately presented and does it cite the current literature?

Yes

If applicable, is the statistical analysis and its interpretation appropriate?

No

Are all the source data underlying the results available to ensure full reproducibility?

Yes

Is the study design appropriate and is the work technically sound?

Partly

Are the conclusions drawn adequately supported by the results?

Partly

Are sufficient details of methods and analysis provided to allow replication by others?

Yes

Reviewer Expertise:

Regenerative Medicine, gene/cell therapy, Muscular Dystrophy, Molecular and cell biology

I confirm that I have read this submission and believe that I have an appropriate level of expertise to confirm that it is of an acceptable scientific standard.

# Response

**Published:** 2024-06-06

**Authors:** Ricardo Adrian Nugraha

**Affiliations:** Airlangga University, Indonesia

Thank you very much for your very constructive feedback.

# Reviewer report: Reviewer response for version 1

**DOI:** 10.5256/f1000research.134822.r243563

**Published:** 2024-02-22

**Authors:** Francesco Galli

**Affiliations:** 1 The University of Manchester, Manchester, England, UK

The authors address an important issue in the heart disease therapy the need of novel approach for the regeneration of damage cardiomyocytes to reduce that factor that might worsen cardiac function.

 The approach of the author looks promising and easy to apply to cells. it addresses both the problem to have an easy and abundant cells' source (using adipose stem cells) and to have an easy protocol of de-differentiation using only one molecule (reversine) instead a cocktail of small molecules.

 While the study is of interest, there are few experimental concerns with this manuscript in its present form.

 1-In figure 1 Author states that only 2% of cells are CD45 and CD35 +. While the figure confirms this data for CD35, for CD45 you can see in the figure around 30-40% of positive cells. How can this result be address? Also expression of DAPI is a key information if the percentage of positive cells needs to be established. 

 2-In figure 2 the author showed by RT-qPCR the expression of Oct4 after treatment with different concentration of REV and at different time. While for the highest concentration of REV40 is possible to observe a trend in the gene expression for the other 2 concentration REV10 and REV20 is observed a random expression during the time course. has the author an explanation for this result? If yes it could be important to be added in the manuscript.

 3-Result in figure 3 is very clear. It well known that downregulation of brachury genes results in down regulation of different mesenchymal marker (e.g. SMA,Vimentin, Desmin) did the author analysis. the expression of this gene? Showing the regulation also of these downstream genes could strength this data and the paper.

 4-How can the author correlate the increase of expression of FLK-1 after a treatment with REV10 with the result obtained for Oct4 gene in figure 1 where the expression was affected only after treatment with REV40?

 5-The level of expressions of GATA4 and cTnT protein are essential to demonstrate the fully differentiation of stem-cell in cardiomyocyte. To strengthen his result the author has to show the immunofluorescence, to observe also if the cells have also the typical cardiac phenotype (expression of sarcomere, and the shape of the cells).

 Author wrote a paragraph about limitations, could be useful for the reader if these limitations are indicated in the results.

Is the work clearly and accurately presented and does it cite the current literature?

Yes

If applicable, is the statistical analysis and its interpretation appropriate?

No

Are all the source data underlying the results available to ensure full reproducibility?

Yes

Is the study design appropriate and is the work technically sound?

Partly

Are the conclusions drawn adequately supported by the results?

Partly

Are sufficient details of methods and analysis provided to allow replication by others?

Yes

Reviewer Expertise:

Regenerative Medicine, gene/cell therapy, Muscular Dystrophy, Molecular and cell biology

I confirm that I have read this submission and believe that I have an appropriate level of expertise to confirm that it is of an acceptable scientific standard, however I have significant reservations, as outlined above.

# Response

**Published:** 2024-03-14

**Authors:** Ricardo Adrian Nugraha

**Affiliations:** Airlangga University, Indonesia

1-In figure 1 Author states that only 2% of cells are CD45 and CD35 +. While the figure confirms this data for CD35, for CD45 you can see in the figure around 30-40% of positive cells. How can this result be address? Also expression of DAPI is a key information if the percentage of positive cells needs to be established. 

 Answer: According to our laboratory analysts, which is supported by Auto Image Analysis Toolbox Software,  immunocytochemistry showed that the DFAT cell population showed positive expression (>95% luminescence of cells per field of view) of CD105 and CD90 and negative expression (<2% luminescence per field of view) of CD45 and CD34.

 2-In figure 2 the author showed by RT-qPCR the expression of Oct4 after treatment with different concentration of REV and at different time. While for the highest concentration of REV40 is possible to observe a trend in the gene expression for the other 2 concentration REV10 and REV20 is observed a random expression during the time course. has the author an explanation for this result? If yes it could be important to be added in the manuscript.

 Answer: Although the expression of Oct4 is reduced with the concentration of Reversine and time extended, the cells had got sufficient differentiation potential. In the concentration REV10 and REV20, Oct4 expression was reduced at the 7 ^th^ day after reversine removal, supporting a possible reversine-induced change in lineage commitment. Moreover, Oct4 signalling is shown to be essential in reversine's activity.

 3-Result in figure 3 is very clear. It well known that downregulation of brachury genes results in down regulation of different mesenchymal marker (e.g. SMA,Vimentin, Desmin) did the author analysis. the expression of this gene? Showing the regulation also of these downstream genes could strength this data and the paper.

 Answer: Yes, in summary, these data clearly suggested that reversine possess a dual activity as it promotes the dedifferentiate into stem-cell-like progenitors, but reduce brachyury expression and results in down regulation of different mesenchymal marker.

 4-How can the author correlate the increase of expression of FLK-1 after a treatment with REV10 with the result obtained for Oct4 gene in figure 1 where the expression was affected only after treatment with REV40?

 Answer: Increase of expression of Flk-1 in DFAT cells indicates the potential of these cells to differentiate into cardiomyocytes. Results suggested that increasing concentration and time extended leads to the increasing level of Flk-1. However, level of Oct4 doesn’t incease, suggesting that mesochymal genes were difficulty up-regualated in this experiments.

 5-The level of expressions of GATA4 and cTnT protein are essential to demonstrate the fully differentiation of stem-cell in cardiomyocyte. To strengthen his result the author has to show the immunofluorescence, to observe also if the cells have also the typical cardiac phenotype (expression of sarcomere, and the shape of the cells).

 Answer: Thank you for your suggestion, authors have added Figure 9 (Immunofluorescence of GATA4 in the cell culture) and Figure 10 (Immunofluorescence of cTnT in the cell culture) to support the results.
